# Integrating culture into primate conservation

**DOI:** 10.1098/rstb.2024.0135

**Published:** 2025-05-01

**Authors:** Patricia Izar, Erica van de Waal, Martha M. Robbins

**Affiliations:** ^1^ Department of Experimental Psychology, Universidade de Sao Paulo, Sao Paulo 05508-030, Brazil; ^2^ Department of Ecology and Evolution, University of Lausanne, Lausanne, Vaud, Switzerland; ^3^ Mawana Game Reserve, Inkawu Vervet Project, Kwazulu Natal, South Africa; ^4^ Department of Primate Behavior and Evolution, Max Planck Institute for Evolutionary Anthropology, Leipzig, Germany

**Keywords:** monkeys, apes, behavioural plasticity, human-induced habitat change, cross-site comparison, cultural evolution

## Abstract

Primates exhibit the richest cultural repertoire among animal taxa, spanning foraging, communication, sociality and tool use. Understanding the cultural behaviours of primates has strongly influenced the study of animal behaviour and challenged traditional views that culture is exclusive to humans. With nearly 60% of primate species endangered owing to human-driven habitat changes, recent calls have emerged to integrate cultural diversity into conservation strategies. However, the integration of culture into primate conservation requires careful planning to avoid misallocation of resources or skewed conservation priorities. Our review reveals that studies on primate culture are limited to less than 3% of extant species, largely owing to taxonomic and methodological biases favouring long-term observations in protected habitats. We propose that including culture in conservation policies can broaden the scope of research, fostering more inclusive conservation agendas that address taxa with diverse habitats and underexplored cultural traits. Furthermore, anthropogenic habitat changes can both erode and foster cultural behaviours, emphasizing the need for context-specific conservation strategies. We suggest that recognizing cultural traits in conservation frameworks may enhance the resilience of primate populations in changing environments. This approach promises a more comprehensive and equitable allocation of conservation efforts, preserving both the biological and cultural diversity of primates.

This article is part of the theme issue ‘Animal culture: conservation in a changing world’.

## 1. Introduction

The first evidence of culture in non-human primates (hereafter, primates) emerged from observations of sweet-potato washing in provisioned *Macaca fuscata* on Koshima Island, Japan, in 1953. Sweet-potato washing has continued to this day as a deeply ingrained tradition in this macaque population. This innovation, based on observations in the field, marked the first discussion about culture in non-human animals, at a time when genetic transmission was the predominant explanation for animal behaviour [1]. Sweet-potato washing behaviour challenged this traditional view by demonstrating that cultural transmission through social learning could occur in non-human primates, thus laying the foundation for the study of animal culture [2,3]. Interestingly, this first observation of cultural behaviour in a wild primate originated from the anthropogenic modification of the habitat, through the provisioning of novel food.

Research on primates has played a pivotal role in shifting the longstanding view that culture is exclusive to humans, paving the way to the recognition of culture across animals. After this first description, the field of animal culture burgeoned, remarkably so in the past 20 years, demonstrating that social animals from insects to apes rely on socially learned behaviours to a much greater extent than previously thought [4]. Primates are interestingto examine cultural variation because they are prone to social learning, they usually live in social groups and are long-lived. Indeed, many reviews have been published—especially focusing on the great apes [5], but also in Catarrhini [3] and Platyrrhini monkeys [6] demonstrating that primates exhibit a wide range of cultural behaviours in diverse domains such as foraging, tool use, sociality and communication (electronic supplementary material, table S1). The cultural behaviours described in the wild probably represent only the tip of the iceberg regarding the diversity of cultural traits exhibited by natural populations [7].

Recently, there has been growing attention to cultural variation both as a justification and an occasion to assist in conservation efforts [8–10]. Primates represent both a cautionary tale and an opportunity when examining the relationship between culture and conservation. They are a cautionary tale because most primate species face the key conservation threats of habitat loss, hunting, illegal trade, invasive species and disease outbreaks, leading to *ca* 60% of species currently facing the risk of extinction [11]. Therefore, we may lose the possibility to understand the behavioural and cultural diversity exhibited as we lose habitat, populations and species [12–14]. Paradoxically, anthropogenic impacts provide the opportunity for primates to adapt to rapidly changing environments, wing to their behavioural plasticity and their ability for social learning and innovation, which may also increase cultural variation [15,16]. The transformation of the environment caused by human activities can thus lead either to the loss of cultures or to an increase in behavioural variation that can be a durable cultural change or just an ephemeral response [15]. Some argue that socially transmitted innovation will only increase fitness in environments with sufficient spatial or temporal stability [6]; otherwise, it could be outdated or inappropriate [17]. However, human habitat modifications can lead to detrimental changes for primates such as poorer health conditions [18] and less time socializing [19].

In this article, we propose strategies for integrating cultural variation into primate conservation, drawing attention to points that require caution; otherwise, efforts and financial resources may be allocated in a biased or misguided manner. First, in §2, we demonstrate that the identification of cultural traits in primates is concentrated in less than 3% of extant species owing to disparities in research agendas, both in terms of field study duration and methodologies used. Thus, based on our current state of knowledge, the integration of cultural variation as a conservation criterion for primates would encompass a very limited number of taxa. On the other hand, emphasizing socially inherited behavioural variation as a conservation criterion could stimulate field research on this topic and support more comprehensive conservation policies that include marginalized taxa with wide distributions and resilient populations that exhibit significant cultural diversity. Second, in §3, we provide some examples of how anthropogenic disturbances, typically viewed as threats to species conservation and even to behavioural variation, can influence primate cultural variation in both directions, either reducing or increasing the number of cultural traits. In §4, we bring together the arguments presented in §2 and §3 to formulate recommendations for the incorporation of culture into primate conservation policies.

## 2. Cultural variation in primates: taxonomic and methodological biases

If we are striving to integrate culture into conservation strategies, having an improved understanding of the amount of cultural variation in primates would be beneficial. One key consideration is the imbalance among primate taxa in research focus on identifying cultural variants. There are currently 719 primate species and subspecies recognized by the IUCN, yet cultural variation has been reported in only 21 species (electronic supplementary material, table S1).

In great apes, there is evidence of cultural variation across all three genera (electronic supplementary material, table S1), including the quantification of cultural variants within different domains for each genus, leading to the identification of 39 cultural variants in *Pan*, 26−35 in *Pongo* and 23 in *Gorilla* [5]. A recent review identified cultural variation in vocal and gestural communication in *Pan troglodytes* (eight variants), *Pan paniscus* (two variants), *Pongo* (four variants) and *Gorilla* (one variant) [20].

In Catarrhine monkeys, there is evidence of cultural variation for two species of *Macaca* (electronic supplementary material, table S1). There is variation across populations/groups in tool use for *M. fascicularis* [21], and in stone handling [22,23] and in food items and feeding techniques for *M. fuscata* [1,2]. In addition, cultural differences in types of interactions with humans were reported in *M. fascicularis* that live in temples [24]. In *Papio*, changes to a peaceful social style following high mortality of adult males have been considered cultural transmission of social behaviour in *P. anubis* [25]. In the genus *Chlorocebus*, cultural variation has also been reported in neighbouring groups of wild *Chlorocebus pygerythrus* with differences in diet, sociality and communication [26–28].

In Platyrrhine monkeys, evidence is available only for eight species of four genera in two sub-families (electronic supplementary material, table S1). One of the most striking examples comes from *Cebus capucinus*: cross-site comparison studies identified 6 cultural variants in social conventions [29] and 20 cultural variants in food processing techniques [30]. Cultural variation in the use of tools for feeding (both in occurrence/absence and in tool types and forms of use) has also been well documented in the subfamily Cebinae: four species of the genus *Sapajus* [31,32] and in one species of *Cebus* [33]. Furthermore, 22 cultural variants in *Ateles geoffroyi* were identified as related to social behaviours, feeding processing techniques and food items [34,35]. Finally, cultural variation in one communicative pattern has been identified in *Alouatta pigra* [36].

To our knowledge, no cultural behaviour in Lemuriformes has previously been reported, despite social learning abilities found during field experiments conducted on *Eulemur rufifrons* and on *Lemur catta* [37].

The taxonomic imbalance in reports of cultural variation probably reflects general biases in field research effort towards a few species at a few long-term field stations ‘motivated by specific primate attributes rather than aspects of species vulnerability’ [38, p. 286]. Between the years of 2011 and 2015, long-term fieldwork was heavily concentrated on the genera *Pan*, *Gorilla*, *Macaca* and *Alouatta* [39]. The 20 most studied primate species included the great ape species, along with species from the genera *Chlorocebus* and *Macaca*, and *Alouatta* and *Sapajus*.

The taxonomic imbalance is also related to the methods used [40]. While the range of methods used to study culture in primates is large, cultural variation in wild primates is likely to be much greater than currently described, partially owing to the limitation of field methods [7]. In apes [41–44], Cebinae [29,30] and Atelinae [34,35], considerable effort has been invested in cross-site comparisons from long-term research sites to identify behavioural variation not explicitly related to ecological or genetic correlates as indicative of cultural variation. Despite its prevalent use to identify culture in primates, and less so in other taxa, the cross-site comparison method does not provide direct evidence of social transmission [45], which is a necessary condition to attribute culture to a species [6]. Moreover, behavioural patterns can still be considered cultural even if ecological and genetic factors are not entirely excluded, provided that social influences play a role in the acquisition of the behaviour [6,23,46,47]. Indeed, greater environmental variability leads to greater behavioural and cultural traits in *Pan* [48]. Additionally, the distribution of tool use in *Sapajus*—one of the most documented examples of cultural variation in Platyrrhine—is influenced by environmental factors like habitat structure and season [31,33,49].

Strong evidence of social learning comes from one long-term study on the development of stone tool use in immature *Sapajus libidinosus*. Immature individuals are drawn to nut-cracking behaviour by observing and hearing skilled adults use tools, which directs their attention to the tools and nuts, aiding their learning process [50,51]. Similar to human cultures, the tools and artefacts created through traditional use play a role in passing down this behaviour to younger generations [52]. The rarity of this type of evidence also emphasizes the need for considerable field efforts and long-term studies [53].

Some within-site research has revealed group-level cultural variation, which provides more control over genetic and ecological factors than the cross-site comparisons. For example, three neighbouring communities of *P. troglodytes* maintain differences in cultural behaviour—preferred tool type use to crack open nuts—despite frequent immigration of adult females [54]. highlightsFurthermore, long-term research on up to six neighbouring groups of *Chlorocebus*, which comprise one single genetic population and live in overlapping home ranges with similar ecology, highlights behavioural variations across multiple domains in a species without material culture [27,28].

Only very rarely have behavioural innovations and their spread been observed, as in the case of *M. fuscata* washing sweet potatoes or *P. troglodytes* moss-sponging to drink water [55]. Because of the difficulty of observing innovation and social transmission in the wild, experiments have been conducted in several species to test the social learning abilities of primates. Most of these experiments have been conducted in captivity, which provides a highly controlled environment but lacks ecological validity. Here, we highlight only a few experimental studies that have been conducted in the field and relate them to the cultural repertoire of the species. Initially, these experiments were conducted to test whether social learning was present, but more recently the focus has shifted to investigating social learning biases, i.e. who learns from whom. These strategies appear to vary during ontogeny, with infants typically learning exclusively from their mothers (i.e. vertical transmission), juveniles expanding their attention to other group members (i.e. oblique transmission) and a third phase occurring after dispersal, with immigrants learning from residents or residents learning from immigrants (i.e. horizontal transmission) [56]. Studies on wild *Chlorocebus* have shown that these three main pathways of social learning can be experimentally validated. Researchers have found vertical social learning of foraging techniques [57,58] and food choice [59]. Oblique transmission was shown experimentally in learning foraging locations from females [60,61] and foraging techniques from high-ranking individuals [62,63], but this also highlighted the importance of the content and context of actions, with experiments showing payoff-biased learning [61,63]. Finally, horizontal transmission was revealed with immigrant *Chlorocebus* individuals conforming to local food preferences [59], but immigrants were also found to be vectors of novel foraging habits [64].

The methodological bias in describing culture in primates has further implications if we are adopting culture as a criterion for conservation. The presence of experimental evidence for social learning in relatively well-studied species that lack observational reports of unique cultural variants reinforces the argument that the extent of primate cultural variation is likely much under-represented. -to-However, field experiments can be less feasible in endangered species in cases where natural behaviour could be detrimentally modified or there is an increased risk of human-to-primate disease transmission, which thus creates a bias in our knowledge of social learning.

In summary, the taxonomic imbalance in identifying cultural units among primates reflects both disparities in field research effort and variation in the methods used to study culture. Considering cultural units in conservation policies requires academic effort to fill the gaps and allow informed resource allocation (§3). In the following section, we will present some examples of anthropogenic disturbance to primate culture.

## 3. Anthropogenic disturbance of primate cultural variation

Humans have been significantly altering the environment for at least 100 000 years, with a dramatic acceleration in the mid-20th century, leading to direct human influence on over 80% of the land surface in the past two decades [65–67]. Accordingly, there has been growing concern over the increasing anthropogenic pressure on most primate populations owing to urbanization, agriculture, timber extraction, mining or hydroelectric operations, introduction of exotic plant species, forest fragmentation, exposure to waste and climate change [68,69].

Deforestation—a major threat to tropical forests—has caused extensive forest loss, with native ecosystems increasingly replaced by pastures and croplands [70]. Consequently, many primate species have adapted to agroecosystems, where natural habitats are substituted with plantations and livestock [71,72]. Although these environments may provide increased food availability, which is reflected in more stable or growing primate populations compared with the average [72], they also expose primates to significant risks, including environmental hazards, human conflicts and zoonotic diseases [73]. Most accounts of human impacts on primates refer to loss of biodiversity [73], but human-induced habitat change also can alter a wide range of primate behaviour including dispersal patterns, diet, aggressive and affiliative behaviour and even vital rates [74–76]. Building on the idea that habitat modification leads to decreased behavioural diversity, animal cultures might also be threatened. van Schaik [77] frames the ‘disturbance hypothesis’ by predicting how different types of disturbance will, in turn, have a socioecological effect and then a cultural effect. Specifically, an increase in hunting may lead to changes in behaviour, including social interactions that could reduce the opportunity for social learning, whereas habitat fragmentation may reduce the likelihood of diffusion of cultural traits [75]. At its extreme level of habitat conversion, loss of all cultural traits will occur.

### (a) Anthropogenic disturbance can reduce primate behavioural and cultural diversity

In Platyrrhine monkeys, geographical variation and human disturbance have influenced social behaviour in groups of *Alouatta pigra* across different locations in Mexicoo [78]. Negrin and colleagues have reported that groups in larger habitats had twice the behavioural repertoire of those in smaller forests, with fewer high-intensity agonistic interactions in areas with more human disturbance. These differences were likely owing to behavioural plasticity in *Alouatta*, but there may be potential costs associated with this adaptability [78].

Some studies have shown that habitat anthropization might risk *Sapajus*’ material culture. Most records of tool use in *Sapajus* were made in areas of increasing habitat transformation owing to human activities [79,80]. The manipulation of improperly discarded human waste can increase the risk of contamination/disease transmission in *S. libidinosus* individuals that use tools [81]. Remotely sensed imagery analysed land cover and agricultural trends at two sites where *S. libidinosus* use feeding tools [82]. From 1987 to 2017, agriculture increased by over 300%, threatening *Sapajus*’ behavioural traditions owing to erosion and vegetation loss. At one site, 14 years of climatic data revealed that habitat changes negatively impacted nut-cracking behaviour, with rising temperatures linked to fewer fruit-bearing palms, less ground activity and reduced tool use [83]. In this population, immatures slowly learn to use stones as tools to crack open hard foods when influenced by the activity of conspecifics and their artefacts [50,51]. The decrease in nut-cracking may negatively impact this cultural practice in the long term, as immatures will have fewer opportunities to learn the skill. Losing this cultural behaviour could also affect the population’s viability, given that tool use significantly improves diet quality for *S. libidinosus* [84]. Therefore, the nut-cracking tradition of *S. libidinosus* positively impacts their fitness, suggesting a cultural variant that could be considered in conservation policies [10]. Similarly, among the coastal populations of *C. capucinus*, those that use tools to crack open bivalves, crabs and sea almond trees link their foraging activity in the intertidal zone more strongly to the tidal cycle than non-tool using populations [85].

Another example of an anthropogenic threat to the conservation of a primate culture is seen in the tool-using *Macaca fascicularis aurea* in Thailand. Over the past 20 years, a significant increase in human economic activities has negatively impacted these macaques on Piak Nam Yai by restricting their access to the coast owing to dog attacks. In addition, the availability of bivalves that the monkeys harvest with tools has decreased owing to human extraction [86].

Within great apes, *Pan* living in areas with high human disturbance exhibited an 88% reduction in behavioural traits compared with those living with the least amount of human impact, based on what is probably the largest dataset of intraspecific variation in cultural traits, obtained from both long-term observations and camera trap data from 46 locations [14]. Adding new dietary items to their foraging repertoire owing to eating human crops has been documented in great apes [87–89], and in some cases, they modify their activity patterns by doing this at night [90].

### (b) Anthropogenic disturbance can increase primate behavioural and cultural variation

Human-induced habitat modifications also may lead to behavioural innovations, but their long-term fitness consequences remain unknown [15]. A very interesting example of culturally maintained token economy is *M. fascicularis* living on temple grounds in Indonesia [24,91] that spontaneously engage in token-mediated bartering interactions with humans after having stolen objects from them such as phones, glasses or bags. This behaviour only occurred in one population of *M. fascicularis* for at least 30 years, despite many other populations living in temples, leading researchers to conclude that it is a socially facilitated cultural behaviour.

Climate change is leading to more natural disasters that affect primate survival and behaviour. Following a hurricane that caused deforestation, *Macaca mulatta* increased their tolerance and decreased aggression toward other monkeys, facilitating access to scarce shade critical for thermoregulation [92]. Notably, social tolerance predicted individual survival after the hurricane, but not before, revealing a shift in the adaptive function of sociality.

In human-modified habitats, *Alouatta*—a highly folivorous genus that usually obtains protein and energy from microbial gut fermentation of ingested leaves—have adopted novel feeding strategies such as the consumption of bird eggs [93] and cooked meat found on plates or in waste bins [94]. Meat consumption may provide *Alouatta* with essential micronutrients that are scarce in plant foods, but eating meat can impair the digestion of plant foods, potentially leading to negative health effects for these monkeys [94]. Primates consuming human foods also alter their activity budgets: *Trachypithecus leucocephalus* in quality habitats play more [95] and urban *Sapajus* forage more compared with forest-dwelling individuals [96].

Ecological and demographic factors may have driven an isolated population of *Brachyteles hypoxanthus*, a species that is typically highly arboreal, to use the ground increasingly for feeding and socializing over a 40 year period [97]. The rise in both the frequency and variety of terrestrial activities, along with the spread of this behaviour through male-biased social bonds, indicates the establishment of a local tradition. This shift from the vertical niche has disrupted natural demographic processes, led to increased mortality, as well as birth rates, yet the population has continued to grow despite habitat saturation, which could be non-adaptive. However, the resulting overpopulation has ensured the survival of the species after mortality outbreaks caused by drought and yellow fever [97].

In Mexico, primates originally inhabiting tropical forests are increasingly exposed to anthropogenic noises in a matrix of modified landscapes. This context affects the vocal behaviour of *Alouatta paliatta* males that vocalize in response to anthropogenic noises with high sound pressure levels, such as aerial traffic, machinery and traffic. This response, though, varied across individuals and groups and was not explicitly related to ecological or genetic factors [98], suggesting a role of social learning. As in the case of meat eating by *Alouatta*, this putative new tradition might not reflect adaptive plasticity, particularly since it increased physiological stress in these monkeys [99].

The paradoxical relationship between anthropogenic habitat changes and the gain or loss of behavioural innovations and culture in primates is exemplified by a population of *M. fascicularis fascicularis* that had not been observed to use tools during the decade prior to the COVID-19 pandemic. Research and tourism stopped owing to the pandemic; yet in 2023, 17 adults were observed using stones in a rudimentary percussive manner, lifting and dropping them to crack open oysters. This behaviour may have emerged owing to the interruption to tourism, which reduced the food supply typically provided by visitors to the macaques, and it may disappear with the return of human activity [100].

The examples given above show how anthropogenic effects have impacted all taxa of primates across all behavioural domains (foraging, tool use, sociality and communication). While these show the ability of primates to adapt to environmental change and that it is possible to document such changes in short time periods, they also reveal how in many cases they are likely to be detrimental to the primates and/or their relationship with humans, particularly in terms of their diet and/or health.

## 4. Integrating culture into primate conservation—recommendations

To address the premise that animal culture is a unique aspect of biology that is worth conserving in a world of increasing anthropogenic disturbances, we make the following recommendations for integrating animal culture into conservation. These are not necessarily novel, and most are applicable to the conservation of primates and other species as well as other domains of animal behaviour [3,10,101–103].

### (a) Following the theme of this special issue, animal culture should be integrated into conservation efforts for primates

Cultural variation, which is now recognized as part of biological evolution and diversity through non-genetic inheritance mechanisms, has been documented in a limited number of species and it is, therefore, worthy of preservation alongside genetic and ecological variation [9,10]. This entails linking scientific findings with conservation management planning and policy making. Currently, the efforts by researchers to document animal culture far outweigh its integration into conservation efforts. We should be aware that there will not be a ‘one size fits all’ approach for all taxa (primates and beyond) because the main threats to a population or species may have different effects on population viability and response to anthropogenic change. For example, pioneering strategies that are being taken with cetaceans may not be applicable for primates [104]. Furthermore, careful consideration of how to integrate animal culture into conservation strategies is crucial. The ‘conservation value’ of single behaviours needs to be weighed against the behavioural diversity observed in a species in particular populations and across their entire distribution [102]. Additionally, for some endangered species (see electronic supplementary material, table S1), extensive conservation strategies are already in place and adding cultural variation may detract from ongoing activities. To our knowledge, there is only one instance where culture has been integrated formally into a conservation management plan for a primate, with the main goal being to establish a baseline understanding of cultural diversity for western *P. troglodytes* [105,106]. The integration of culture into conservation strategies should rely on evidence-based approaches whose application is feasible and should augment a species’ conservation efforts rather than duplicate existing approaches [102].

### (b) As scientists, we need to move beyond documenting and describing cultural traits and get involved in applied research

This article (and theme issue) is written by academics for a scientific publication, so it is likely to be read almost exclusively by other academics. While this may increase awareness of the topic and hopefully change the research focus of some scientists, it is primarily reaching only half the audience it is intended for (i.e. not conservation practitioners). To date, the main focus of studying animal culture has been from the perspective of the topics of social learning and behavioural plasticity (fundamental science approach), with less emphasis on the consequences of such behaviours in a human-dominated landscape (applied science approach). For example, we may herald the ability of *P. troglodytes* to adapt to living in rural areas and modifying their diet to include human cultivars, but it is imperative that we also address the conservation challenges of these behaviours because they result in the loss of income and food security for local communities, and in some cases, result in human injuries and death [74]. This divide between fundamental and applied research is further emphasized by the lack of scientific evidence for the effectiveness of conservation strategies in the published literature [102].

### (c) Balance research and conservation actions

The challenges faced by academics to be effective in implementing conservation actions are many, including that many of the conservation activities done by scientists working at universities or research institutions are not recognized in a manner that assists in obtaining research funding or professional job security (tenure, promotions, research grants) [101,103]. This may be viewed as a problem of ‘trying to wear too many hats’, but most academic positions are meant to focus not only on research and scholarship but also on teaching and mentoring, both of which can be done on all educational levels, including conservation education activities with communities residing near field sites. More incentives are needed to encourage academics to train students from the regions where primates are native as well as to recognize scholarly activities that go beyond publications [101,103].

### (d) Despite our increasing understanding of primates’ flexibility to adapt to changing landscapes owing to their social learning abilities, we still need to focus on preserving natural habitat

We should be cautious in assuming that primates’ adaptations to human-modified habitat will not have long-term negative consequences (see above). Comparative studies of species in wild, rural and urban habitats will contribute to our understanding of what behaviours change depending on the environment and the mechanisms underlying any changes. As several examples of cultural variation in non-threatened Catarrhine and Platyrrhine monkeys exist (electronic supplementary material, table S1), identifying environmental factors linked to unique cultural practices, such as tool use in *Sapajus* [31,107], can strengthen the case for habitat conservation.

### (e) There is a need for both short-term and long-term studies, which inevitably results in a trade-off of understanding spatial and temporal variability of behavioural plasticity in a changing environment

Long-term field sites have conservation value, particularly in terms of habitat preservation and reduced illegal activities [102], but if such sites are truly research sites, they also must have value in terms of studying life-history patterns for long-lived species, temporal variation in ecology and behaviour, etc. Much of what we know about cultural variation in Platyrrhine taxa and great apes is owing to there being multiple long-term research sites that have revealed such connections between ecological conditions and behavioural patterns (e.g. [39]). *Pan troglodytes* is undoubtedly one of the most studied primate species, and our knowledge of their cultural diversity results from both multiple research sites that have endured for decades [5,38] as well as one of the most geographically extensive short-term studies of *Pan* populations across Africa, the PanAf program [14]. To aim for extensive understanding of cultural variation for other species, in addition to research from long-term sites, it would be beneficial to place more emphasis on short-term studies across larger geographical areas and then strategically decide where to conduct more in-depth studies.

### (f) We should seek to avoid biases towards particular taxa and cultural domains

Studies on primate culture are limited to less than 3% of extant species; therefore, addressing the existing taxonomic bias in primate research and conservation efforts is challenging [37], as understudied species/taxa are likely to receive less conservation attention. To date, a large focus has been on material culture (tool use), which may have a taxonomic bias (see electronic supplementary material, table S1), but it may also result from material culture having physical evidence that can be studied, alongside scientists’ interest in the cognitive skills needed for material culture and cumulative culture. Carvalho *et al.* [102] point out that focusing solely on cultural traits such as foraging behaviour that can be linked to vital rates (survival and reproduction) may lead to an underestimate of the value of social behaviour or communication traits that may be important for patterns of sociality.

## 5. Conclusion

It is increasingly recognized that the future existence of primates will rely not only on conservation efforts in protected areas but also in human-dominated landscapes [74]. Typically, habitat loss or modification was assumed to lead to local loss of primates, but we now know that most species can adapt to modified habitat (e.g. [87]). Furthermore, in addition to traditional means of protection such as law enforcement to reduce illegal killings, changes in practices and policies at all levels of government are needed [108]. Therefore, more effort is needed to understand how primates adapt to changing landscapes, including the loss of cultural traits, innovations and modifications [15], and to communicate this knowledge to local communities and policymakers for integration into conservation management plans that promote primate–human coexistence [109]. This will necessitate improved dialogue between scientists and conservation managers, harmonization with other conservation activities and strategic activities on local, national and international scales. These recommendations are a tall order, as is the challenge of reducing the ongoing extinction crisis. Overall, it is necessary to put the ‘culture as a conservation strategy’ in perspective in terms of effort and funding so that we do not overemphasize the importance of studying cultural diversity while populations continue to decline. We cannot let the library burn as we are counting the books [110].

## Data Availability

Supplementary material is available online [111].
